# Difficult-to-Treat Skin and Soft Tissue Infections Caused by Panton-Valentine Leukocidin-Producing Community-Associated Methicillin-Resistant *Staphylococcus aureus*: A Case Series

**DOI:** 10.3390/idr17060137

**Published:** 2025-11-03

**Authors:** Luca Pipitò, Chiara Vincenza Mazzola, Giulio D’Agati, Eleonora Bono, Raffaella Rubino, Silvia Bonura, Claudia Gioè, Teresa Fasciana, Antonio Cascio

**Affiliations:** 1 Department of Health Promotion, Mother and Child Care, Internal Medicine and Medical Specialties “G D’Alessandro”, University of Palermo, 90133 Palermo, Italy; chiaravincenza.mazzola@community.unipa.it (C.V.M.); giulio.dagati@community.unipa.it (G.D.); eleonora.bono@community.unipa.it (E.B.); teresa.fasciana@unipa.it (T.F.); 2 Infectious and Tropical Disease Unit, Sicilian Regional Reference Center for the Fight Against AIDS, AOU Policlinico “P. Giaccone”, 90127 Palermo, Italy; raffaella.rubino@policlinico.pa.it (R.R.); silvia.bonura@policlinico.pa.it (S.B.); claudia.gioe@policlinico.pa.it (C.G.); 3 Antimicrobial Stewardship Team, AOU Policlinico “P. Giaccone”, 90127 Palermo, Italy; 4 Microbiology and Virology Unit, AOU Policlinico “P. Giaccone”, 90127 Palermo, Italy

**Keywords:** community-associated MRSA, community-acquired MRSA, PVL, Panton-Valentine leucocidin, methicillin-resistant *Staphylococcus aureus*, oritavancin, dalbavancin, long-acting agents

## Abstract

**Background**: Community-associated methicillin-resistant Staphylococcus aureus (CA-MRSA) has emerged as a genetically distinct lineage from healthcare-associated MRSA (HA-MRSA), often producing Panton-Valentine leukocidin (PVL) and causing severe skin and soft tissue infections (SSTIs) in otherwise healthy individuals. **Methods**: We describe five cases of PVL-positive CA-MRSA SSTIs admitted to the Infectious Diseases Unit of the University Hospital “Paolo Giaccone,” Palermo, Italy, between 2024 and 2025. Case inclusion followed the CDC criteria for CA-MRSA. Microbiological identification was performed using MALDI-TOF mass spectrometry, and antimicrobial susceptibility testing followed EUCAST standards. PVL gene presence was confirmed by polymerase chain reaction. **Results**: Clinical management included surgical drainage, systemic antibiotic therapy, and decolonization of both patients and close contacts. Long-acting lipoglycopeptides (oritavancin or dalbavancin) were evaluated as therapeutic options to achieve clinical resolution. **Conclusions**: PVL-positive CA-MRSA infections are characterized by recurrence, intrafamilial clustering, and frequent therapeutic failure with standard oral agents. Effective management requires an integrated approach combining prompt surgical drainage; systemic therapy, preferably including long-acting lipoglycopeptides; and comprehensive decolonization of all close contacts.

## 1. Introduction

Community-associated methicillin-resistant Staphylococcus aureus (CA-MRSA) emerged in the 1990s as a lineage genetically distinct from healthcare-associated MRSA (HA-MRSA). CA-MRSA exhibits significant genetic heterogeneity: the USA300 clone is the most frequently reported worldwide, whereas ST80-IV was the first identified in Europe. Initially described as a pathogen responsible for community-acquired infections in otherwise healthy individuals, CA-MRSA strains have progressively spread into hospital settings, leading to nosocomial infections now defined more by genotype than by epidemiological criteria [1,2,3].

Several molecular techniques have been used to characterize CA-MRSA, including pulsed-field gel electrophoresis, multilocus sequence typing, spa typing, and SCCmec typing [2]. Typically, CA-MRSA is associated with Panton–Valentine leukocidin (PVL) production and shows phenotypic susceptibility to tetracycline, clindamycin, and trimethoprim–sulfamethoxazole (TMP-SMX) [1]. Nearly all MRSA strains carry the SCCmec element, which encodes the low-affinity penicillin-binding protein 2a responsible for β-lactam resistance. The type IV SCCmec element, strongly associated with CA-MRSA, lacks genes conferring resistance to non–β-lactam antimicrobials [4]. Conversely, HA-MRSA strains are generally resistant to these agents and do not produce PVL [2].

PVL exerts cytotoxic effects on polymorphonuclear leukocytes, monocytes, and macrophages, leading to complement-mediated cell lysis and the release of pro-inflammatory cytokines [5]. Its presence has been linked to greater virulence and increased mortality [5]. Furthermore, some strains possess the arginine catabolic mobile element (ACME), which enhances virulence and skin persistence [4,6]. CA-MRSA isolates display greater virulence and biological fitness than traditional HA-MRSA strains [6]. Consequently, CA-MRSA is considered more aggressive than HA-MRSA and has been implicated in severe invasive diseases, including community-acquired pneumonia, endocarditis, osteomyelitis, necrotizing fasciitis, and difficult-to-treat skin and soft tissue infections (SSTIs), usually occurring in immunocompetent adults without classical risk factors [4,7].

In vitro studies suggest that clindamycin, linezolid, and fusidic acid may inhibit PVL production, whereas β-lactams—through penicillin-binding protein 1—may paradoxically enhance PVL expression by modulating sarA and rot regulators [5]. Recurrent SSTIs represent the clinical hallmark of PVL-producing CA-MRSA. Lesions are often extensive, and transmission risk increases in contexts of close personal contact [5]. Skin lesions typically present with erythema, swelling, pain, and purulent exudate [4]. Nonetheless, *S. aureus* frequently colonizes human skin and mucous membranes (nares, pharynx, skin, perineum) asymptomatically [8]; therefore, decolonization strategies in carriers are essential to prevent transmission [5].

Despite growing awareness of PVL-producing MRSA as a public health concern, current epidemiological data remain limited both in the Palermo area and across Italy. Previous studies have demonstrated a heterogeneous and polyclonal MRSA population, with ST22 (EMRSA-15) as the predominant clone and sporadic detection of ST8, ST1, and ST398. More recent reports confirm this polyclonality, with no single dominant PVL-positive clone identified to date [9,10,11,12,13,14,15,16].

Here, we report five cases of difficult-to-treat cutaneous infections caused by PVL-producing CA-MRSA in previously healthy individuals (Table 1). Additionally, one previously published case of sexually transmitted CA-MRSA was included for comparison and discussion [17].

## 2. Materials and Methods

We described five cases of CA-MRSA infection admitted to the Infectious Diseases Unit of the University Hospital “Paolo Giaccone,” Palermo, Italy, between 2024 and 2025.

According to the Centers for Disease Control and Prevention (CDC) definition of CA-MRSA infection, patients were included if they met the following criteria:

- A positive MRSA culture result obtained as an outpatient or within 48 h of hospital admission;
- No permanently implanted medical devices or indwelling catheters;
- No previous history of MRSA infection;
- No recent hospitalization or residence in a nursing home or long-term care facility [18].

For each case, PVL toxin was detected by polymerase chain reaction (PCR). The detection of the PVL genes (lukS-PV and lukF-PV) was performed using an in-house PCR assay amplifying a 433 bp overlapping fragment and using primers previously described in the literature. The primers used were: PVL-F (forward) 5′-ATCATTAGGTAAAATGTCTGGACATGATCCA-3′ and PVL-R (reverse) 5′-GCATCAACTGTATTGGATAGCAAAAGC-3′ (expected product ≈ 433 bp) [19].

Identification of the microorganisms isolated from clinical specimens was routinely performed using matrix-assisted laser desorption/ionization time-of-flight (MALDI-TOF) mass spectrometry (MALDI Biotyper, Bruker Daltonics, Billerica, MA, USA).

Antimicrobial susceptibility testing was carried out using an automated system (Phoenix, Becton Dickinson Diagnostics, Sparks, MD, USA), and minimum inhibitory concentrations (MICs) were interpreted according to EUCAST 2025 breakpoints, see Table 2 [20].

## 3. Results

### 3.1. Case 1

A previously healthy 19-year-old male presented to the emergency department with fever, thigh pain, asthenia, and purulent skin lesions. His past medical history was notable only for amoxicillin allergy. He was a soccer player and denied illicit drug use. Eight days before admission, he developed right inguinal lymphadenopathy and erythematous papules on the lower limbs, likely following minor trauma sustained during a soccer match. He self-administered ibuprofen without improvement. Lesions worsened, leading to phlegmon formation and fever onset.

**Figure 1:**
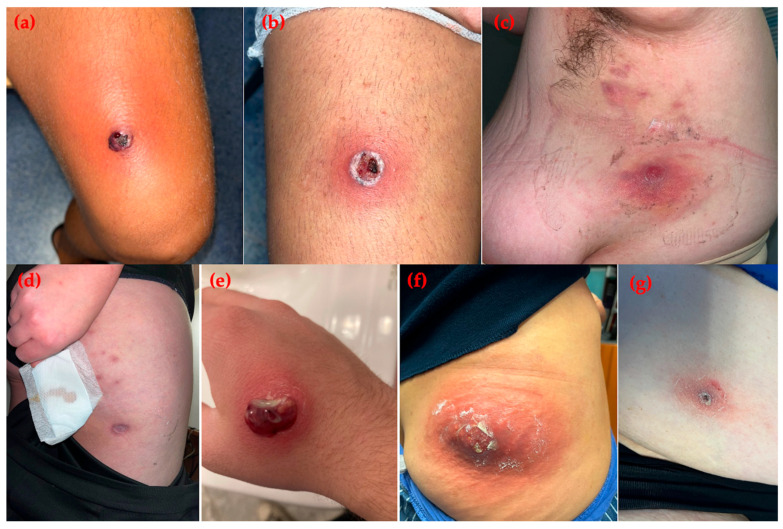
Skin and soft tissue infections caused by community-associated methicillin-resistant Staphylococcus aureus. (**a**,**b**) Case 1: purulent lesions on the lower limbs of a young soccer player; (**c**,**d**) Case 2: nodular and pustular lesions on the subaxillary region and thigh in an obese woman from a familial cluster; (**e**) Case 3: single purulent lesion on the right hand of an adolescent male; (**f**) Case 4: carbuncle on the right buttock of a female patient; (**g**) Case 5: crusted lesion on the lower limb of a comorbid female patient.

Upon admission to the Infectious Diseases Unit, vital signs were as follows: temperature 38 °C, heart rate 80 bpm, respiratory rate 20 breaths/min, blood pressure 110/70 mmHg, and oxygen saturation 80% on room air. Physical examination revealed three phlegmonous lesions with purulent discharge (Figure 1a,b). Laboratory findings showed leukocytosis (WBC 14,290/µL; normal 4000–11,000/µL), neutrophilia (94.3%; normal 40–74%), and elevated C-reactive protein (CRP 121 mg/L; normal < 5).

Chest and abdominal computed tomography (CT) scans were unremarkable, whereas lower-limb CT revealed soft tissue involvement with three fluid collections (right: 4 and 9 mm; left: 15 mm). Screening swabs for MRSA and carbapenemase-producing bacteria were negative, as was HIV testing. Blood cultures and lesion swabs were obtained. Empirical therapy with daptomycin (10 mg/kg/day) plus meropenem (1 g every 8 h) was initiated. Due to persistent fever and lesion progression, surgical drainage was performed on day 2. Lesion swabs yielded MRSA, whereas blood cultures remained sterile. Phenotypic susceptibility indicated community-acquired MRSA, and PVL genes were detected by PCR. Meropenem was discontinued, and intravenous fosfomycin (4 g every 6 h) was added to daptomycin. After surgical drainage, the patient’s condition rapidly improved, with resolution of fever. Transthoracic echocardiography excluded endocarditis or cardiac dysfunction. Following seven days of intravenous therapy, he was discharged with an additional seven-day oral linezolid regimen. No relapse or reinfection was observed during 12-month follow-up.

### 3.2. Case 2

A 26-year-old obese woman presented with recurrent skin lesions. Her past medical history was otherwise unremarkable. Multiple family members (father, mother, two sons, sister, and nephew), including the patient herself, were involved in a cluster of PVL-positive CA-MRSA infections. Her sons had previously been hospitalized with impetiginous lesions on the face, scalp, axillae, hands, and feet. The patient presented with nodules and pustules on the lower limbs and axillae (Figure 1c,d). Vital signs were normal. Laboratory tests were unremarkable except for mildly elevated CRP (7.4 mg/L; normal < 5). Blood cultures were negative, but nasal swabs tested positive for MRSA. She had previously received topical antibiotics followed by oral clindamycin (450 mg every 8 h) and TMP-SMX (160/800 mg every 12 h) for seven days. Decolonization with chlorhexidine baths and intranasal mupirocin was advised for all household members. After one month, both the patient and one son relapsed. Given the recurrent nature of infection and failure of oral therapy, intravenous oritavancin was administered. During infusion, she developed chills that resolved after slowing the infusion rate, with no further adverse effects.

The patient reported that her husband had not undergone decolonization because he was asymptomatic. A new decolonization cycle, including the husband, was therefore recommended. No immediate relapses occurred, although mild recurrences developed two months later, resolving spontaneously or with topical treatment. After one year, reinfection manifested as a subnuchal abscess requiring drainage and oritavancin therapy in the emergency department. No microbiological samples were collected during this episode.

### 3.3. Case 3

A 14-year-old male was admitted for recurrent purulent skin lesions for several months, unresponsive to multiple oral antibiotic regimens, including amoxicillin–clavulanate and topical gentamicin. His medical history included recurrent pharyngitis since early childhood, with the last episode occurring 10 months earlier.

On admission, he was afebrile and hemodynamically stable. Physical examination revealed purulent lesions and phlegmons on the right hand, arm, and trunk (Figure 1e). Laboratory tests showed leukocytosis (WBC 14,510/µL; normal 4000–11,000/µL), neutrophils 70.8% (normal 37.5–77%), and elevated CRP (35.8 mg/L; normal < 5). Lesions were drained manually, and cultures grew CA-MRSA. PVL genes were confirmed by PCR, and nasopharyngeal swabs were MRSA-positive.

The patient received intravenous dalbavancin (1000 mg), achieving complete clinical resolution. Mild recurrences occurred in the following months, resolving with topical therapy, and no further hospitalizations were needed.

### 3.4. Case 4

A 43-year-old woman presented with a painful, purulent lesion on the right buttock that had progressively worsened over two weeks despite oral TMP-SMX and was accompanied by fever. Her past medical history included asthma and a prior perioral *S. aureus* infection. Her partner also reported recurrent pustular lesions.

On examination, a painful carbuncle (≈15 cm) with purulent discharge and surrounding erythema was observed (Figure 1f). Vital signs were normal. Laboratory findings revealed WBC 11,050/µL, neutrophils 79.7% (normal 40–74%), CRP 41.7 mg/L (normal < 5), and presepsin 226 pg/mL (normal <200). Surgical drainage was performed, and cultures grew PVL-positive CA-MRSA. Blood cultures and nasal swabs were negative. Transthoracic echocardiography and pelvic MRI excluded endocarditis and deep abscesses.

The patient received intravenous oritavancin (1200 mg) along with surgical drainage. Decolonization with mupirocin and chlorhexidine was recommended for her and the partner. Two months later, she developed mild left-buttock cellulitis with fever. Oral doxycycline failed, suggesting reinfection with CA-MRSA. Intravenous dalbavancin (1500 mg) was administered, followed by repeat decolonization, which was successful.

### 3.5. Case 5

A 61-year-old woman with type 2 diabetes mellitus, obesity, hypertension, and dyslipidemia presented with recurrent cutaneous lesions. In December 2024, she developed impetiginous, exudative lesions on the abdomen and right shoulder, initially treated with topical antibiotics without improvement. New lesions appeared on the abdomen and lower limbs despite dermatologic follow-up and additional topical therapy (Figure 1g).

Microbiological samples collected during an infectious disease consultation yielded CA-MRSA susceptible to tetracyclines and TMP-SMX. She received oral TMP-SMX for three weeks with partial benefit, followed by two weeks of oral linezolid, resulting in incomplete resolution. A decolonization regimen with intranasal mupirocin and chlorhexidine washes was also performed.

Due to persistent lesions, she was admitted to our department for intravenous oritavancin (1200 mg) and a renewed decolonization protocol. Examination revealed multiple crusted lesions on the abdomen and lower limbs. Vital signs and laboratory tests were within normal limits. Both nasal and lesion swabs grew PVL-positive CA-MRSA. The patient achieved a favorable clinical response.

## 4. Discussion

CA-MRSA has become a major global cause of SSTIs, with PVL-positive strains particularly associated with tissue necrosis, abscess formation, and recurrent infection. PVL-induced injury results from the uncontrolled release of proteases by lysed neutrophils, while host defense mechanisms include neutralizing antibodies and serum protease inhibitors [21]. Unlike HA-MRSA, CA-MRSA predominantly affects otherwise healthy individuals. In sub-Saharan Africa, where PVL is endemic, the high incidence of SSTIs among young patients has been linked to immature humoral immunity and the consequent absence of protective antibodies [22].

Our case series provides an updated picture of this phenomenon in Italy, where PVL prevalence remains relatively low [8,9,10,11,12,13,14,15,16]. Nevertheless, familial and community clusters underscore the pathogen’s capacity for rapid dissemination. The cases in our cohort spanned a broad age range, from adolescence (Case 3, 14 years) to older adulthood with comorbidities (Case 5, 61 years), supporting existing evidence that CA-MRSA infections are not limited to specific demographic groups. Athletic activity was implicated in Case 1, consistent with the literature linking CA-MRSA to contact sports such as football, where minor skin trauma, turf burns, and close physical contact increase transmission risk [23,24]. Sexual transmission was suspected in Case 6, previously described by our group [17]. Similar reports have documented the circulation of CA-MRSA within MSM networks [25].

Household clustering was evident in Cases 2 and 4, where incomplete or refused decolonization contributed to relapse. In Case 2, the husband’s refusal of decolonization preceded reinfection, while Case 4 involved recurrent lesions in both partners. Interfamilial outbreaks in Italy have been described by Rimoldi et al. [16], who reported identical PVL-positive clones among household members. Nasal colonization was most common, with occasional rectal carriage [16].

All patients presented with recurrent or complicated SSTIs caused by PVL-positive CA-MRSA, highlighting key clinical aspects. Manifestations were consistent with previous reports: recurrent abscesses, deep pyoderma, and extensive purulent lesions. Prior studies demonstrated that PVL-positive isolates are more frequently associated with purulent SSTIs, recurrence, and need for surgical drainage compared with PVL-negative strains [26,27]. Although our series did not include invasive infections, the literature describes severe presentations such as osteomyelitis, bacteremia, and septic shock, sometimes linked to zoonotic or environmental exposure [7,24,28].

Treatment remains challenging because CA-MRSA often recurs despite targeted therapy. Relapses arise from persistent colonization of patients and contacts, environmental contamination, and the microorganism’s ability to develop resistance and tolerance. Asymptomatic colonization of the nares, groin, or axillae and contamination of household surfaces facilitate reinfection even after apparent cure [29,30]. Transmission through fomites and contaminated surfaces further complicates eradication and promotes recolonization [23]. Public environments, such as restrooms, gyms, beaches, and public transport, as well as personal objects (mobile phones, coins, currency, bedding) may act as reservoirs [23].

In our cohort, nasal colonization was detected in all but Case 1, although other body sites were not assessed.

Decolonization is crucial to prevent relapse, yet success rates are modest (<50%) and influenced by patient comorbidities [31]. We did not formally evaluate decolonization outcomes, but adherence appeared suboptimal in some cases. Both patients and household members should adopt strict hygiene measures [31]. A multicenter randomized controlled trial in cystic fibrosis patients showed that early MRSA eradication therapy combining oral antibiotics (TMP-SMX or minocycline plus rifampin), topical decontamination (nasal mupirocin, chlorhexidine body wash), and environmental cleaning achieved an 82% eradication rate versus 26% with observation alone (*p* < 0.001) [32]. Sustained negativity persisted in about half of the treated patients through day 84 [32]. However, evidence on decolonization efficacy in CA-MRSA carriage remains limited. Topical therapy alone is effective mainly for isolated nasal colonization [33]. Combining intranasal mupirocin with chlorhexidine body wash is the most reliable approach for community carriers, whereas adding systemic antibiotics may improve eradication in extra-nasal carriage [33]. In our series, all patients except Case 1 underwent decolonization, but probable non-compliance led to relapse in Cases 2 and 4. Although global mupirocin resistance is increasing, none of our isolates displayed resistance [34].

Antibiotic management of CA-MRSA SSTIs remains debated. Mild cases, as in Cases 2 and 3, sometimes resolve spontaneously through host immune clearance and can be treated with topical agents alone. In immunocompetent patients with small abscesses (<5 cm), incision and drainage represent first-line therapy and may suffice for healing, though adjunctive antibiotics can reduce recurrence in selected cases [35,36]. Studies have shown comparable failure rates between oral therapy (TMP-SMX or clindamycin) and drainage alone [37]. Current guidelines recommend systemic antibiotics for severe, extensive, or rapidly progressive infections; cases with systemic symptoms or immunosuppression; lesions in difficult-to-drain sites; or failure of drainage alone [30,31].

Vancomycin remains a standard option but is limited by dosing complexity and nephrotoxicity [3,31,38]. Protein synthesis inhibitors such as clindamycin and linezolid, which also suppress toxin production, are valuable alternatives [31]. Surgical source control is nevertheless essential [31]: four of our patients required drainage (Cases 1–4), confirming its central role in recovery. Oral agents (TMP-SMX, clindamycin, doxycycline) were ineffective despite in vitro susceptibility (Cases 2, 4, 5), emphasizing the virulence of PVL-positive strains and the limits of pharmacologic therapy without proper source control.

Our experience also highlights the potential utility of long-acting lipoglycopeptides in refractory or recurrent CA-MRSA infections. Oritavancin and dalbavancin, used in four patients (Cases 2–5), reduced hospitalization and improved adherence compared with prolonged intravenous therapy. Mild recurrences occurred in Cases 2 and 3 but resolved without additional systemic treatment. Tolerability was excellent, with only mild infusion-related symptoms in one case. Reinfection occurred one year later in Case 2 and was again successfully treated with oritavancin, illustrating both the challenge of complete eradication and the need for long-term follow-up. Case 4 experienced a reinfection a few months later, which required a second course of long-acting therapy.

These findings support long-acting lipoglycopeptides as valuable options for recurrent infections, particularly in patients with comorbidities, poor adherence, or oral therapy failure. Nevertheless, relapses can occur even after long-acting treatment, underscoring the need for standardized management protocols.

A limitation of this study is the absence of molecular typing of isolates. Consequently, we cannot determine whether infections were due to previously circulating clones or sporadic introductions. Despite this, our cases provide meaningful clinical insights into the management of recurrent CA-MRSA and reinforce the need for ongoing molecular surveillance to guide epidemiologic monitoring and infection-control strategies.

## 5. Conclusions

PVL-positive CA-MRSA infections are characterized by recurrence, intrafamilial clustering, and frequent therapeutic failure with standard oral agents. Effective management requires an integrated approach combining prompt surgical drainage; systemic therapy, preferably including long-acting lipoglycopeptides; and comprehensive decolonization of all close contacts.

These cases demonstrate that even in non-endemic regions, PVL-positive CA-MRSA should be suspected in patients presenting with recurrent purulent SSTIs. Successful management must address both individual treatment and prevention of household transmission through coordinated clinical and infection-control measures.

## Figures and Tables

**Table 1:** Features and outcome of patients with community-associated methicillin-resistant *Staphylococcus aureus*.

Age (years) and Sex	Risk Factor	Comorbidity	Clinical Features	Previous Oral Therapy	Therapy and Duration	Relapse Requiring Hospital Admission
Case 1. 19, M	Athletic	None	Fever and purulent skin lesions with exudation on the lower limbs	None	Daptomycin plus fosfomycin (7 days) and switch to oral linezolid (7 days)	None
Case 2. 26, F	Familial cluster	Obesity	Nodules and pustules on lower limbs and axillae	Clindamycin and TMP-SMX	Oritavancin 1200 mg single dose	Yes
Case 3. 14, M	None	Recurrent pharyngitis	Purulent lesions and phlegmons on the right hand, arm, and trunk	Amoxicillin/clavulanic acid	Dalbavancin 1000 mg single dose	None
Case 4. 43, F	Partner with skin lesions	None	Fever and carbuncle on the right buttock	TMP-SMX	Oritavancin 1200 mg single dose	Yes
Case 5. 61, F	None	Diabetes mellitus, obesity, hypertension, dyslipidemia	Crusted lesions on the abdomen and lower limbs	TMP-SMX and linezolid	Oritavancin 1200 mg single dose	None
Case 6. 40, M *	Multiple instances of unprotected heterosexual intercourse	None	Pustules and nodules on the back. Previous lesion on the penis	None	TMP-SMX (7 days)	None

* The case was previously described and published [17].

**Table 2:** Antibiograms: susceptibility and (MIC) of community-associated methicillin-resistant *Staphylococcus aureus* isolates.

Antibiotic	Case 1	Case 2	Case 3	Case 4	Case 5	Case 6 *
Fusidic acid	R (8)	R (8)	S (≤0.5)	S (≤0.5)	R (8)	S (≤0.5)
Ceftaroline	S (0.5)	S (0.5)	S (1)	S (0.5)	S (0.5)	S (0.5)
Ciprofloxacin	I (≤0.5)	I (≤0.5)	I (≤0.5)	I (≤0.5)	I (≤0.5)	I (≤0.5)
Clindamycin	S (≤0.25)	S (≤0.25)	S (≤0.25)	S (≤0.25)	S (≤0.25)	S (≤0.25)
Daptomycin	S (≤0.5)	S (≤0.5)	Not tested	S (≤0.5)	S (≤0.5)	S (≤0.5)
Erythromycin	S (≤0.25)	S (≤0.25)	R (>2)	S (≤0.25)	S (≤0.25)	R (>2)
Gentamicin	R (>4)	R (>4)	S (≤1)	R (>4)	R (>4)	S (≤1)
Linezolid	S (2)	S (2)	S (1)	S (2)	S (2)	S (1)
Mupirocin	S (≤256)	S (≤256)	S (≤256)	S (≤256)	S (≤256)	S (≤256)
Oxacillin	R (>2)	R (>2)	R (>2)	R (>2)	R (>2)	R (>2)
Penicillin G	R (>0.25)	R (>0.25)	R (>0.25)	R (>0.25)	R (>0.25)	R (>0.25)
Teicoplanin	S (≤0.5)	S (≤0.5)	S (≤0.5)	S (≤0.5)	S (≤0.5)	S (≤0.5)
Tetracycline	S (≤0.5)	S (≤0.5)	S (≤0.5)	S (≤0.5)	S (≤0.5)	R (>2)
Tigecycline	S (≤0.25)	S (≤0.25)	S (≤0.25)	S (≤0.25)	S (≤0.25)	S (≤0.25)
Cotrimoxazole	S (≤1/19)	S (≤1/19)	S (≤1/19)	S (≤1/19)	S (≤1/19)	S (≤1/19)
Vancomycin	S (1)	S (1)	S (1)	S (1)	S (1)	S (1)

* The case was previously described and published [17]. S: susceptible, I: susceptible, increased exposure; R: resistant.

## Data Availability

No new data were created or analyzed in this study. Data sharing is not applicable to this article.

## Abbreviations

The following abbreviations are used in this manuscript:

CA-MRSA	Community-associated methicillin-resistant *Staphylococcus aureus*
HA-MRSA	Healthcare-associated MRSA
PVL	Panton-Valentine leukocidin
PCR	Polymerase chain reaction
TMP-SMX	Trimethoprim/sulfamethoxazole
SSTIs	Skin and soft tissue infections
